# How Specific Abilities Might Throw ‘*g*’ a Curve: An Idea on How to Capitalize on the Predictive Validity of Specific Cognitive Abilities

**DOI:** 10.3390/jintelligence6030041

**Published:** 2018-09-07

**Authors:** Matthias Ziegler, Aaron Peikert

**Affiliations:** Psychological Institute, Humboldt-Universität zu Berlin, Unter den Linden 6, 10099 Berlin, Germany; aaron.peikert@hu-berlin.de

**Keywords:** *g*-factor, specific abilities, scholastic performance, school grades, machine learning, curvilinear relations, ability differentiation

## Abstract

School grades are still used by universities and employers for selection purposes. Thus, identifying determinants of school grades is important. Broadly, two predictor categories can be differentiated from an individual difference perspective: cognitive abilities and personality traits. Over time, evidence accumulated supporting the notion of the *g*-factor as the best single predictor of school grades. Specific abilities were shown to add little incremental validity. The current paper aims at reviving research on which cognitive abilities predict performance. Based on ideas of criterion contamination and deficiency as well as Spearman’s ability differentiation hypothesis, two mechanisms are suggested which both would lead to curvilinear relations between specific abilities and grades. While the data set provided for this special issue does not allow testing these mechanisms directly, we tested the idea of curvilinear relations. In particular, polynomial regressions were used. Machine learning was applied to identify the best fitting models in each of the subjects math, German, and English. In particular, we fitted polynomial models with varying degrees and evaluated their accuracy with a leave-one-out validation approach. The results show that tests of specific abilities slightly outperform the *g*-factor when curvilinearity is assumed. Possible theoretical explanations are discussed.

## 1. Introduction

Scholastic performance is an important predictor of later academic success [1,2], health [3], and job success [4]. Moreover, many decisions are based on the grades students achieve in school (e.g., college or university admission). It is therefore not surprising that research has focused its attention on grades. One study by French, Homer, Popovici and Robins [4] looking at educational attainment and later success in life specifically targeted high school GPA as a predictor, recognizing it as an important predictor of later job success. It is also just consequential that much research has been devoted to the predictors of scholastic success, mostly using grades as dependent variable e.g., [5,6,7,8]. Here, as in many other fields [9,10,11,12,13], general mental ability or the *g*-factor has often been singled out as the best predictor of scholastic performance [14,15,16] with specific abilities purportedly adding little or no explained variance [17]. However, this focus on the so-called general factor seems to not take full advantage of the structure of intelligence [18,19], which postulates a hierarchical structure with a multitude of specific abilities located at lower levels beneath a *g*-factor. This hierarchical structure also finds support in brain research [20]. However, research focusing on the predictors of scholastic success now often takes *g* for granted and only looks at other constructs to improve predictions [21,22,23,24]. The current special issue, as well as this paper, aims at reviving the debate about specific cognitive abilities and their importance for scholastic success. Building on the concepts of criterion deficiency and contamination [25] as well as on the theory of ability differentiation [26,27], we suggest two mechanisms which both would lead to curvilinear relations between specific abilities and grades. The disregard of such nonlinear models in the past might have yielded the wrong impression about the fidelity of specific abilities with regards to school grades. Additionally, we will also test the idea of such curvilinear relations using a machine learning approach.

### 1.1. Critique on the g-Factor and Its Use as Single Predictor of Performance

Much of the research on the *g*-factor covers areas outside of the scholastic domain, focusing on academic performance, health, job performance, or longevity. Thus, the critique on *g* also originates from a diverse set of researchers. Possibly the most fundamental critique can be found in studies doubting the sheer existence of *g*. Kovacs and Conway [28]. For example, it was postulated that cognitive tests require several processes and that an overlap of such processes might be the cause for finding *g*. This view has been harshly refuted e.g., [29]. Others have stressed that the strong emphasis on *g* has hindered progress on research of intelligence in its breadth [30]. Schneider and Newman [31] reviewed the literature and suggested six reasons why lower order factors of cognitive ability need to be considered despite the often reported importance of *g*. Their first statement is to point out that the empirical fit of hierarchical and multifactor models of intelligence often outperforms the fit of unidimensional models [32,33,34]. A fly in the ointment is the low construct reliabilities often observed for scores reflecting specific abilities once the *g*-factor is controlled for [35,36]. This can be seen as a first hint that gains from looking at specific abilities cannot be huge. In fact, Schneider and Newman use these small but nevertheless verifiable incremental contributions as their second reason to further investigate specific cognitive abilities. A meta-analysis by Ziegler et al. [37] looking at the predictive validity of specific ability tests with regards to job training also reports such low but significant values when not considering the principle of compatibility or level of symmetry [38,39]. This principle of compatibility also stated by Schneider and Newman means that predictor and criterion need to have a similar level of symmetry or level of abstraction to find optimal correlations. Thus, specific abilities might be better suited to predict specific performance [40]. Ziegler et al. [37] also showed this for the prediction of training success and motoric abilities. Lang et al. [41] reported similar findings with job performance as the criterion. Another reason for focusing on specific abilities stated by Schneider and Newman are the sound theoretical models and empirical studies supporting the notion of a broad spectrum of second order abilities [19]. Another argument is that the effect of adverse impact can be ameliorated by differentially weighing specific cognitive abilities. Finally, Schneider and Newman point towards bifactor models as a possible means to better gauge the effects of specific abilities. While all of these arguments are compelling and important, with one exception, they all focus on the predictor side, meaning cognitive ability. Only the compatibility principle also integrates the criterion side. In the case of scholastic performance, especially when operationalized via grades, it seems worthwhile though to consider both the criterion and the predictor side.

### 1.2. Considering the Criterion—Specific Ability Relations

One of the more obvious problems with grades is criterion contamination and deficiency [25,38]. Grades can be derived from a variety of different exam forms: written tests, oral tests, or active participation to name just a few. Even when restricting research to written tests only, there are tests that are more objective (e.g., math exams) and tests that are less objective (e.g., essays) in a psychometrical sense. Thus, grades can be contaminated, which means that the variance of grades might in part be due to aspects actually not reflecting scholastic performance e.g., gender [42]. Criterion contamination could also mean that some of the criterion variance is actually not due to differences related to the predictor side. Considering that scholastic performance requires a multitude of abilities, traits, and skills [7], it is absolutely reasonable to assume that cognitive abilities will not be related to all psychological processes contributing to scholastic performance. In that sense, grades are contaminated with variance unrelated to cognitive abilities. We will come back to this notion later on.

On the other hand, cognitive abilities encompass such a broad spectrum of different processes, e.g., [31,43], that it is unlikely that school grades are influenced by all of these processes. This in turn can be regarded as a criterion deficiency. Unfortunately, considering contamination and deficiency has so far only been used to explain lower test criterion correlations in general. Thus, to revive specific abilities as predictors, these ideas need to be considered in a new way. We will do this here by connecting them with other influences on test-criterion correlations.

**Variance decomposition and compatibility.** Above, we have already mentioned one such influence: the compatibility aspect. So far, we have only looked at it in terms of level of symmetry or specific context. Unfortunately, it is impossible and maybe not even desirable to change grading to be better compatible with ability tests. There is, however, also a modeling perspective. Most of the research on intelligence and scholastic performance uses an average of grades in which each grade contributes equally. This is especially puzzling as research shows that the relation between grades and intelligence varies with subject [16]. Thus, when a trivial scoring function like the average is used, the variance shared by all grades is most likely being maximized due to the variance sum law (see Equation (1)). This is similar to a *g*-factor score, which maximizes the shared variance of the used ability tests. Clearly, this might favor the *g*-factor as predictor. However, even in studies where latent variables are used to decompose variance, specific abilities often do not contribute to performance. The alleged advantage of latent variable modeling is that each indicator of a reflective latent variable only contributes with the variance actually reflective of this variable, thereby decomposing the indicator variance into its constituents. The latent variable embodies shared variance and the residuals of the indicators specific variance. Deary et al. [44] used such latent variable modeling to estimate the true score correlation between scholastic performance and *g* (which was 0.81). However, this model would also have allowed testing for specific relations between residuals on both sides. This means that residuals of specific abilities (after controlling for *g*) could have been correlated with residuals of specific subject performance (after controlling for GPA). If such model modifications were to achieve a better model fit, it would show that specific ability test scores do in fact contain relevant variance. The reported model fit (CFI = 0.92) suggests room for model improvement [45,46]. Following up on this idea seems promising. However, this only makes sense with data that allow for modeling specific abilities such as latent variables, which means that item level information would be required. Unfortunately, the data set provided for analyses for all papers published in this special issue did not contain such fine-grained data. Thus, we will not report our findings here, but will provide details in material provided on the OSF link to this paper.^1^ We report there how, by using variance decomposition based on structural equation modeling, we aimed at identifying specific relations, which might be blurred when using test scores or grades comprising different variance sources. In that sense, the relations between the residual factors were meant to have better compatibility and thus better chances of finding optimal correlations [39].

**Nonlinear relations between cognitive ability and school performance.** Another idea, which can be pursued with the current data, regards the nature of the relation between predictor and grade. As is typical in psychology, prior research almost exclusively used correlations and regressions, thereby assuming linear relations between predictors (abilities) and criteria (scholastic performance). However, following up this line of thinking would mean that more intelligence always yields better performance. This idea also means that the relation between ability and performance is assumed to be consistent across all levels of the predictor and criterion. Psychologically, this would mean that ability always contributes in the same way to performance. We have already pointed out above that scholastic performance is made up of a multitude of psychological processes. For example, when learning about linear algebra, it is certainly important to bring fluid and crystallized abilities along to understand the topic. At the same time, research shows that acquiring knowledge also benefits from being open to new stimuli and being interested in them [47], having a mastery oriented learning style [7], and a specific motivational structure [21,22]. Each of these traits points towards specific processes. Some of these are about allocating energy to learning and others are more about the persistence with which goals are pursued. Importantly, all of these contribute to scholastic performance incrementally to *g*. Coming back to the notion of criterion contamination, these relations can be considered contamination from the perspective of cognitive abilities as predictors. Thus, the changing importance of other predictors could be one mechanism causing curvilinear relations between ability and performance. Thus, for easy or moderately difficult tasks, cognitive ability could be relevant only to get a first understanding while motivation and learning style might be more important to obtain excellent results. For more complex tasks, it could be that differences in intelligence play out especially in the upper performance regions where rote learning and motivation alone are no longer sufficient. Consequently, it is reasonable to assume that believing in a linear world is highly problematic. Instead, it seems more than justified to also consider curvilinear relations. Before further discussing aspects of curvilinear relations, we want to introduce a second, and related mechanism which would lead to curvilinear effects, the ability differentiation hypothesis.

### 1.3. Considering the Predictor—Ability Differentiation Hypothesis

Spearman [26] is often credited for introducing the idea of differentiation within the structure of cognitive abilities across different ability levels [27]. Spearman, in his famous two factor theory, assumed that underlying all cognitive tasks is a general factor which is then complemented by a specific ability. Spearman also assumed that the role of this *g-*factor and the respective specific ability might differ across ability levels (He assumed a similar mechanism across age, which has found little empirical support though [48,49]). The core idea of this so-called ability differentiation hypothesis is that the role of the *g-*factor varies across different ability levels. In that sense, *g* can be seen as some kind of fuel for all specific abilities. Consequently, if the amount of fuel and in psychological terms, if central processes are limited, all other processes relying on this capacity will also be limited [50]. Higher ability groups in turn have more fuel and thus fewer limits on general capacity. Thus, specific abilities can play a more important role. Deary et al. [51] reasoned that only sufficient *g* would provide the foundation for applying specific abilities. As one consequence, the *g* factor has more saturation in lower ability groups. Using moderated factor analysis, such an effect could be confirmed in large and heterogeneous samples [48,49]. Moreover, there is also meta-analytical support for the notion of ability differentiation [52]. Importantly, this also bears consequences for the current research project. If we consider that sufficient *g* provides the foundation to apply specific abilities, we could expect that the relation between specific abilities and cognitive tasks cannot be linear. Only with increasing capacity with regards to *g* would specific abilities have sufficient fuel to influence task success. At the same time, higher *g*-factor levels are very likely to go along with higher scores on specific ability tests. Now, if we consider what we just pointed out above, which is the notion that tasks might require cognitive abilities depending on their level of complexity, we could reason that these effects can only be realized by students with sufficient *g*. Importantly, this would also lead to curvilinear relations between specific abilities and grades. To give a hypothetical example, in order to understand the mechanisms of linear algebra, numerical abilities might be needed in addition to *g* to master the basic performance level. In order to solve more complex tasks, often vested in short texts, verbal abilities might come into play and be even more important. As a consequence, the relation between numerical ability and performance would be stronger for grades reflecting low to moderate performance. Verbal abilities in turn might have stronger relations in the upper performance level. In each case, curvilinear relations would occur, suppositional on sufficient *g*-factor scores. Considering that samples like the one used here have undergone years of schooling and thus years of selection, it seems reasonable to assume that *g*-factor scores suffice. Considering the *g*-factor as the fuel or foundation for the specific abilities would also mean that it is predictive throughout the grade range, which would result in a more linear prediction. We want to stress here that this is purely speculation based on different theories; thus, we will not propose explicit hypotheses regarding the relation between specific abilities and specific subjects.

### 1.4. Curvilinear Relations

**Figure 1:**
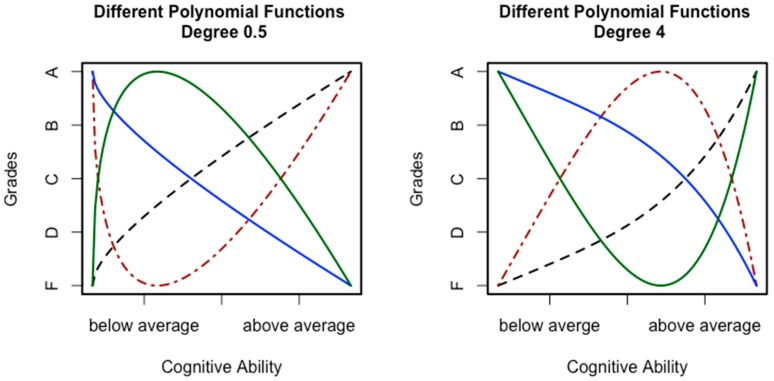
Hypothetical examples for curvilinear relations between cognitive ability and scholastic performance.

Curvilinear relations are comparable to a moderation effect. Whereas a moderation means that the slope representing the relation between two variables is influenced by a third variable, a curvilinear relation means that the slope is influenced by the level of one of the variables [53]. In that sense, the variable itself is the moderator. For the present research question, the relation between cognitive abilities and grades, this would mean that this relation depends on the level of the predictor. In other words, increments in ability are no longer contributing in the same way as before. Two general kinds of such curvilinear relations are reasonable when looking at the relation between cognitive ability and grades (see Figure 1). The first kind (see the left-hand side of Figure 1) would assume a negatively accelerated relation. This means that the relation between grades and ability is more pronounced in the lower ability regions. Importantly, the relation could be positive or negative in general. Moreover, it is also possible that the relation actually reverses at a certain point. A classical example for such a relation is the Yerkes–Dodson law [54]. A more relevant and more recent example can be found in a study by Antonakis et al. [55]. Those authors reported an inverted u shape relation between cognitive ability (measured with the Wonderlic test [56]) and perceived leadership behavior. Considering the relation between cognitive ability and grades, it seems unlikely that higher ability could actually decrease performance. However, it seems reasonable to assume that, from a certain point on, the impact of cognitive ability on performance might weaken or reach a plateau. An example for such a relation can be found in the study by Ganzach et al. [57]. Those authors could show that the relation between general mental ability and pay follows a curvilinear trend. For grades, this would mean that being brighter only gets you part of the way, which is one of our key arguments explained above. Afterwards, other aspects might be more important for achieving good grades. In that sense, grades would be contaminated as explained above.

On the right side of Figure 1, a positively accelerated relation is depicted. The general idea here is that the relation between ability and performance starts out weak and then increases with increasing ability. As before, a reversal in the relation is also possible. Such a relation signifies the idea that cognitive abilities become important only at a higher level of ability and likewise when it is about better grades. This idea is in line with the ability differentiation hypothesis. This hypothesis assumes that specific abilities are more important at higher *g* levels. Here, it is important to note that *g* and the specific abilities are positively correlated. Thus, it is reasonable to assume that higher levels for specific abilities go along with higher *g* levels. For grades, this could mean that, due to having fewer limits with regards to general processes [50], specific abilities are able to exert their influence without restrictions. Especially for exams requiring solving complex problems, this should be advantageous, leading to better grades. Thus, we would expect stronger relations in the upper specific ability levels. 

The different functions depicted in Figure 1 all have in common that the relation between ability and grades is not linear, or in other words, ability does not contribute equally across the whole ability range. We have argued above that this might be due to other predictor–performance relations also influencing performance. Importantly, these other relations might include subject specific aspects (e.g., the teacher or difficulty of exams). This can be explained with trait activation theory [58], which assumes that differences due to a trait manifest depend on the situation. Amongst other ideas, trait activation theory proposes the existence of constraints and distracters, which are situational features that make the manifestation of a trait less likely [59]. Such constraints and distracters might differ across subjects, which means that they would not contribute to the correlation between grades in all cases. Thus, when averaging across subjects, the specifics that led to a changing impact of ability will become relatively less important. This is due to the fact that adding variances also means to add the correlation between the summands:

(1) $$\mathrm{Variance}\;\mathrm{Sum}\;\mathrm{Law}:\;\sigma_{x\pm y}^{2}=\sigma_{x}^{2}+\sigma_{y}^{2}\pm 2\rho_{xy}\sigma_{x}\sigma_{y}.$$

In Equation (1), *ρ* equals the population estimate of the correlation and *σ* represents the population estimate of the standard evaluation. Thus, as mentioned before, it is vital to establish an equal level of symmetry. Therefore, we will analyze the impact of cognitive abilities on grades separately for each school subject.

It is now feasible to relate these statistical ideas to the ideas of criterion contamination and deficiency, as well as to the ability differentiation hypothesis. The first mechanism, suggested above, is based on criterion deficiency and puts forward the idea that specific abilities are more or less important at different grade levels. At other grade levels, the psychological processes underlying those specific abilities are less relevant and the grade is deficient in the sense that its variance is not due to the variance reflecting the specific ability. The variance is also contaminated because it contains the influences of other, most likely non-cognitive, traits. The second mechanism refers to ability differentiation and assumes that specific-abilities can only exert their influence at higher ability levels.

Another open question, however, is what specific kind of curvilinear relation to assume.

### 1.5. Modeling Curvilinear Effects

When it comes to modeling curvilinear effects, quadratic functions [60] seem to dominate psychological literature. While this kind of relation is intuitive, the modeling also comes along with a number of problems. On the very top of the problem list probably is the need for large samples due to lower power to detect such curvilinear effects [61]. Another issue is the threat of exploiting common method bias. This threat, however, has been refuted by Siemsen et al. [62]. One of the more severe criticisms is that a quadratic relation just is one out of many possible curvilinear relations [63]. Finally, it has recently been suggested that exploring the assumption of a simple quadratic relation (u or inverse u-shape) with linear regressions can be misleading [64]. Here, we will try to use a different statistical approach.

From a theoretical point of view, the idea of a changing relation between ability and grades might intuitively best be captured by a quadratic function. However, there is no convincing argument that a different curvilinear function would not be even better suited. In general, such functions are called polynomial functions. The term with the highest power (exponent) determines the degree of the polynomial. According to Cortina [53], it is vital to include nonlinear as well as linear terms. Thus, a simple function assuming only one predictor and the corresponding linear and curvilinear terms would be:

(2) $$y_{i}=x_{i}+x_{i}^{p}+\varepsilon_{i}.$$

In this equation, *x* represents the predictor and *y* the criterion value for person *I*; *p* represents the degree of the polynomial. The last term reflects an individual prediction error (ε). It would be easy to look at the relation between ability and grades only with such an equation and only with one value for *p* (e.g., with *p* = 2). However, it seems much more promising to test several possible values for *p* and to select the best fitting out of all models. In machine learning, the use of complex algorithms to model relations is very common [65]. In that sense, multiple models with different values for the parameters are tested and a best fitting model is selected. This selection is then validated using different approaches. The field of psychology has been rather reluctant to embrace the chances that machine learning offers [66], the needed sample size probably ranging amongst the most important barriers. However, Yarkoni and Westfall [67] just recently propagated the use of machine learning in psychology, especially when aiming at prediction (also see [68] for an applied example). Considering the relation between grades and ability and its intricate complexities, machine learning seems like a promising avenue. While we applaud the recommendation to use machine learning in general, we will not apply complex algorithms like support vector machines or deep neural networks here for two reasons. The first reason is a statistical one. Machine learning results are prone to overfitting, which means that the complex algorithms find relations that are hard to replicate. Typically, this is dealt with during the validation by approaches in which data are split several times or hold-out samples are used. We will also use this approach here but still make a note of the relatively small sample size. The second reason is a more psychological one. Machine learning is often criticized as yielding black box algorithms that cannot be understood. While this might be true for very complex approaches, less complex approaches yield results that still can be interpreted straightforwardly. One such approach very useful for modeling curvilinear relations are complex polynomial functions [63]. The idea is to test different possibilities for *p* and then selecting the function yielding the best result. This function can then also be used to interpret the underlying processes. Clearly, this is a totally exploratory approach. Thus, the results should be considered as possibilities or hypotheses. The need for independent replications is especially high here. 

In general, a polynomial function with linear and nonlinear terms and several predictors is:

(3) $$y_{i}=\beta_{0}+\beta_{1}x_{i,1}+\beta_{11}x_{i,1}^{p_{1}}+\beta_{2}x_{i,2}+\beta_{22}x_{i,2}^{p_{2}}+\cdots +\beta_{q}x_{i,q}+\beta_{qq}x_{i,q}^{p_{q}}+\varepsilon_{i},\;i=1\ldots n\;q=1\ldots k$$

As before, *x* reflects the predictor and *y* the criterion values for person *i*. The second index, *q*, refers to the number of the predictor. In our case, when using specific abilities, we will have three different predictors. The power *p* reflects the degree of the polynomial. The index for each of these *p* values reflects the idea that the degrees of the polynomials can vary across predictors. In the current example, this would mean that the type of curvilinear relation between each specific ability test score and grades is not fixed but can be different. Finally, the impact of each term on the criterion is reflected in the regression weight *β*.

### 1.6. Summary and Aims of the Study

Considering the importance of school grades for success in later life, identifying the predictors of scholastic performance and shedding light onto the nature of these specific relations is an important goal. With regards to cognitive abilities as predictors, prior research has often emphasized the importance of *g* and questioned the use of specific cognitive abilities. The current paper proposes the idea of curvilinear relations between specific abilities and grades based on the idea of criterion contamination and deficiency as well as on the ability differentiation hypothesis. To be more precise, we propose that performance on different levels, meaning different grades, requires different abilities. For example, whereas numeric abilities might be necessary to master the basics of a new mathematical technique, verbal abilities might come into play at higher levels. Moreover, at specific levels of performance, other traits, for example, personality, interests, and motivation, might be more decisive, thereby causing a change in the impact of ability. Another mechanism could be that, due to ability differentiation, specific abilities can only exert their impact on performance in higher ability ranges, thereby also resulting in better grades. Considering Spearman’s ideas about the *g*-factor, an impact of these mechanisms on the relation between grades and the *g*-factor is less likely. Thus, the test-criterion relations of specific abilities should profit more from adding curvilinear terms.

The current paper aims at testing the idea of curvilinear relations between specific abilities and grades. To this end, we will use an easy machine learning approach based on polynomial regressions. The advantage of such an approach is that more complex relations than just quadratic relations can be tested as well. This procedure must be considered data driven and exploratory. Thus, we will not propose any specific hypotheses and content ourselves with finding the best models for each school subject and then coming up with post hoc hypotheses as to the nature of the effects. We hope that these hypotheses as well as the statistical approach in general can inspire future research. 

## 2. Methods

### 2.1. Sample, Measures, and Procedure

The data set used here is the one provided by the guest-editors of this special issue. It contains a sample of *n* = 219 students (*n* = 117 females) with a mean age of 16.03 (*SD* = 1.49). Each student value on the specific ability tests Unfolding, Analogies, and Number Series was reported. Unfolding is a figural test from the Wilde-Intelligence Test 2 [69]. Both of the other tests are from the same test battery. Analogies is a verbal analogy test and number series a complex test, which is why it is still considered an indicator of reasoning. Additionally, grades in math, German, English, and physical education (sports) were provided. The grades were coded with a range from 1 to 6 (from 1 = insufficient, 2 = with deficits, 3 = sufficient, 4 = satisfactory, 5 = good, 6 = very good). No further details were provided. Descriptive statistics as well as correlations between all variables can be found in Table 1. 

### 2.2. Statistical Analyses

All analyses were conducted in R [71] using RStudio [72] and the packages *psych* [73], *lm.beta* [74], *knitr* [75], *apaTables* [76], *caret* [77], *tidyverse* [78], and *readr* [79]. In order to prepare the data, we first estimated a *g* score for each person by extracting a factor score on the first unrotated factor of a factor analysis (principal axis factoring) of the three specific ability tests. The loadings were 0.57 (Unfolding), 0.58 (Analogies), and 0.68 (Number Series). The eigenvalue was 1.13 and the factor explained 38 percent of the variance. In a next step, we scaled the predictors (i.e., the three specific ability tests as well as the factor score) to use them in the polynomial regressions [56]. To avoid nonessential collinearity resulting from scaling [80] and, in order to ease interpretation, we centered the scores. We also followed advice form machine learning literature and additionally standardized the scores [80]. We used a value of three as a center and a variance of one. The value of three was chosen to avoid negative values, which are not defined in some polynomials (e.g., *p* = 0.5). Moreover, the grade scale theoretically ranges from 1 to 5, which makes 3 the theoretical mean value.

In a next analytical step, we simply ran two multiple linear regressions for each school subject. Within the first regression, we used the factor score representing *g* as independent variable. In the second regression, the three specific ability test scores served as independent variables. These analyses were conducted as a kind of baseline for the following analyses.

We then ran two series of polynomial regressions for each school subject. In the first series, we used the factor score as a proxy for *g* as predictor. In the other series, we used the three specific ability test scores. In order to avoid collinearity, we did not include the *g*-factor score in the specific ability models. We also refrained from residualizing the *g*-factor score in order to keep interpretability straightforward. In order to compare the *g*-factor models with the specific ability models, we compared the prediction accuracy and the adjusted *R*^2^s. While the prediction accuracy tells us something about the utility of the model in general, the *R*^2^ helps to gauge the effect size. In a regression analysis, the variance shared by all predictors with the criterion is reflected in *R*^2^. Thus, larger values for the specific ability models would imply an additional impact of the specific ability test scores compared to the *g*-factor only models. In each series, we ran a sequence for *p* starting at a value of 0 to a value of 5 in steps of 0.5. We left out the value of 1 because it would lead to collapsing the terms meant to represent linear and nonlinear aspects. We did allow the *p*-values to vary for each predictor (resulting in 8000 models for each grade and predicted by the specific abilities). Thus, the functions were:

(4) $$grade=\beta_{0}+\beta_{1}\cdot g+\beta_{11}\cdot g^{p}+\varepsilon ,$$

for the *g*-factor score and:

(5) $$grade=\beta_{0}+\beta_{1}\cdot Unfolding+\beta_{11}\cdot Unfolding^{p_{1}}+\beta_{2}\cdot Analogies+\beta_{21}\cdot \;Analogies^{p_{2}}+\beta_{3}\cdot Number\;Series+\beta_{31}\cdot Number\;Series^{p_{3}}+\varepsilon ,$$

for the specific abilities, respectively. In order to minimize the risk of overfitting, we used a leave-one-out technique. In particular, we ran each possible model on the whole data set, leaving out one person. The resulting function was used to predict the value of this left out person. This was repeated for every person in the data set. The absolute values of the differences between the actually observed grades and the predicted values were then computed, yielding 219 deviations. These values followed a skewed distribution (many small deviations, few large ones), which is why we decided to use the median and not the mean of these values. This median was used as an estimate of the prediction accuracy (labeled RMSE (root mean square error) following the tradition in the literature) and the model with the lowest value was selected. In that sense, the selected model is the one yielding the lowest discrepancy between the observed and the model implied grade. Using this approach, we selected the best model for each grade and each predictor combination (i.e., *g*-factor score vs. specific ability test scores). In order to decide whether the models with the specific ability test scores yielded larger effect sizes for a specific grade, we also compared the respective adjusted *R*^2^s. For each selected model, we used the *R*^2^ estimated using the selected function and data from the complete sample.

To get a better idea of the function behind the models for the specific abilities, we plotted the functions varying each predictor and holding the other predictors constant at the centered mean value. Thus, for each grade and the specific ability test score models, three functions were plotted. All R codes and results (as html) can be found in the OSF material.^2^

## 3. Results

### 3.1. Multiple Linear Regressions

Table 2 contains the results for the linear regressions using the *g*-factor score as independent variable. It can be seen that the models yield moderate (German and English) to strong relations (math) with the exception of sports. Accordingly, the regression weights were significant with the exception of sports. Table 3 contains the findings obtained when using scores for the three specific abilities as predictors. While the *R*^2^′s are descriptively larger for the models with the specific ability test scores, the adjusted *R*^2^′s are mostly smaller. The exception here is English, where specific ability test scores achieve a larger *R*^2^. However, the difference is only 0.7 percent. Again, sports could not be predicted at all. The regression weights for the Analogies score were significant for math, German, and English. The Unfolding and Number Series scores only predicted math grades. Thus, all in all, the classical approach does not yield findings in support of using specific ability test scores. Moreover, at this point, we decided to drop the sports grade from further analyses.

### 3.2. Selecting the Best Fitting Model

**Figure 2:**
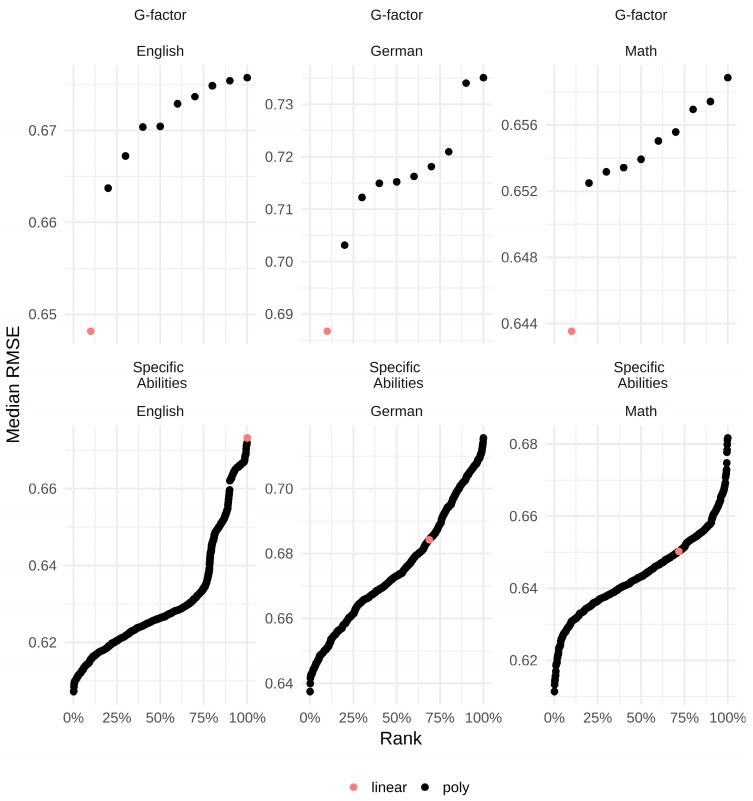
Ranked RMSEs for all models in ascending order. Percentiles on the *x*-axis. A red dot always represents the model with linear terms only.

In the next step, we tested the different polynomial regression models, thereby testing curvilinear relations. Figure 2 contains the RMSEs for all models with the *g*-factor only and all models with the specific ability test scores as predictors in ascending order.

**Figure 3:**
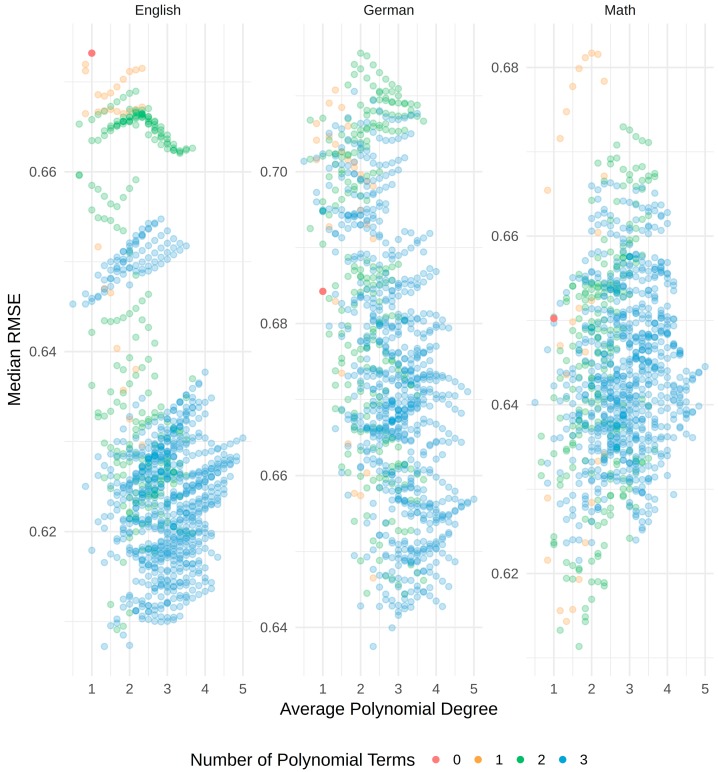
RMSEs for all models. The *x*-axis lists the average of the polynomial degrees (e.g., Unfolding^2, Analogies^3, NumberSeries^4 results in an *x*-value of [2 + 3 + 4]/3 = 3). The model with linear terms only is depicted in red.

As can be seen, the values roughly range between 0.60 and 0.70. Thus, the differences between the models were not pronounced. Moreover, it can be seen that, while the model with linear terms only fit best whenever the *g*-factor score was used as a predictor, the models with specific ability test scores as predictor yielded the best results when assuming curvilinear relations. In order to exemplify the influence of changing from a linear to a curvilinear model, Figure 3 depicts the RMSEs for all models specified for each grade and in relation to the average polynomial degree as well as the actual number of polynomial terms. The different colors reflect the number of polynomial terms ranging from zero (linear model) to 3 (all specific ability test scores have a curvilinear relation with the grade). To simplify the figure, the actual polynomial degrees were averaged. Those values are used on the *x*-axis. The *y*-axis reflects the RMSE of each model. For example, the plot on the right contains all RMSEs for models with math grade as dependent and specific ability test scores as independent variables. It can be seen that the model with linear terms only (red dot) ranged in the middle. Thus, there were many curvilinear models better, but also many worse than the linear model for math as dependent variable. It can further be seen that the best models for math had two polynomial terms whose average degrees were below 2 (green dots). However, those models also belonged to the worst models for math, which indicates that the three specific ability test scores might have quite dissimilar relations with the grades. Regarding the other grades, the linear models never performed well.

The parameter estimates for the best models can be found in Table 4. For the *g-*factor models, this was the linear model in all cases. For the models with the specific ability test scores as predictors, the results were different. Here, the polynomial achieved more accurate predictions (lower average RMSEs) and we selected them as the best fitting models. Looking at the actual polynomial degrees shows that only the Unfolding and Analogies test scores had curvilinear relations with the grade in each school subject. The Number Series test score did not have a curvilinear relation with the math grade.

When comparing the *g*-factor only and specific ability test score models for each school subject, it can be seen that the models with the specific ability tests scores yielded a more accurate prediction (lower average RMSE) for all grades. Importantly, these advantages in accuracy, which might simply be due to the larger number of predictors, were also reflected with regards to the adjusted *R*^2^s: models with the specific ability test scores and curvilinearity were better for all subjects. Referring to Gignac and Szodorai [81], the effects can be considered as small to medium (also see [82]). Comparing the differences with the differences between the adjusted *R*^2^′s from the linear regressions (see Table 3 and Table 4) shows that the predictive power of the specific ability test scores profited relatively strongly, now yielding an advantage compared with the *g*-factor score models.

All in all, the results support the notion of curvilinear relations between specific cognitive ability test scores and grades.

### 3.3. Exploring the Nature of the Curvilinear Relations

To explore the nature of the found relations, we plotted the best fitting models for all specific ability test score models and the curvilinear *g*-factor model. As could be seen in Table 4, the *g*-factor score always has a linear relation with the grade. The Number Series scores (blue line) also followed an almost linear relation. For Analogies scores (green line), the nature of the curve suggests an accelerating relation. This would mean that the captured ability has a stronger relation with performance for higher levels of this ability. This was especially pronounced for the math grade. Unfolding scores (orange line) had a curvilinear relation with all grades. In each case, its relation deteriorated around the mean level.

## 4. Discussion

The current paper addressed the issue of specific vs. general cognitive abilities by referring to three theoretical ideas. First, we emphasized the necessity to match predictor and criterion in terms of the level of abstraction (comparability principle). Second, we suggested two mechanisms, which would impact the form of the relation between specific ability test scores and grades. We referred to Brogden’s [25] ideas of criterion contamination and deficiency to deduce a mechanism influencing the relation between specific ability test scores and performance. We assumed that the influence of specific abilities changes once other traits become more or less important for performance. The other, suggested mechanism, building on Spearman’s ability differentiation hypothesis, was the idea that specific abilities exert their influence only when sufficient levels are reached. Both mechanisms would yield nonlinear relations between the specific abilities and scholastic performance. This was the third theoretical idea brought forward and tested here using the provided data set. Applying polynomial regressions, it was found that models containing linear and nonlinear terms outperform simple linear models when using specific ability test scores. Moreover, these models with specific ability tests were more accurate and better predictors of grades compared to models only containing a factor score reflecting *g.* Finally, the analyses showcase the utility of machine learning.

### 4.1. Specific Abilities and Scholastic Performance

As was reported before, using scores from specific ability tests to predict scholastic performance did not yield findings superior to using a *g*-factor as the only predictor when using multiple linear regressions. However, the picture changed, when polynomial regressions were used and thus when assuming curvilinear relations. In particular, while this did not change the predictive power of *g*, the findings for the specific ability tests were improved. In fact, the improvement was strong enough to tentatively state that the models outperformed both the linear models as well as the models only containing *g*. While this finding is interesting per se, it also bears some potential theoretical implications. Especially with regards to the scores obtained from the Unfolding and Analogies tests, the findings call for more specific hypotheses. Above, we have already stated that we will not make specific a priori hypotheses. Now that we know the results, we will suggest some post hoc explanations based on these findings. We want to emphasize that these explanations are totally data driven at this point. 

**Figure 4:**
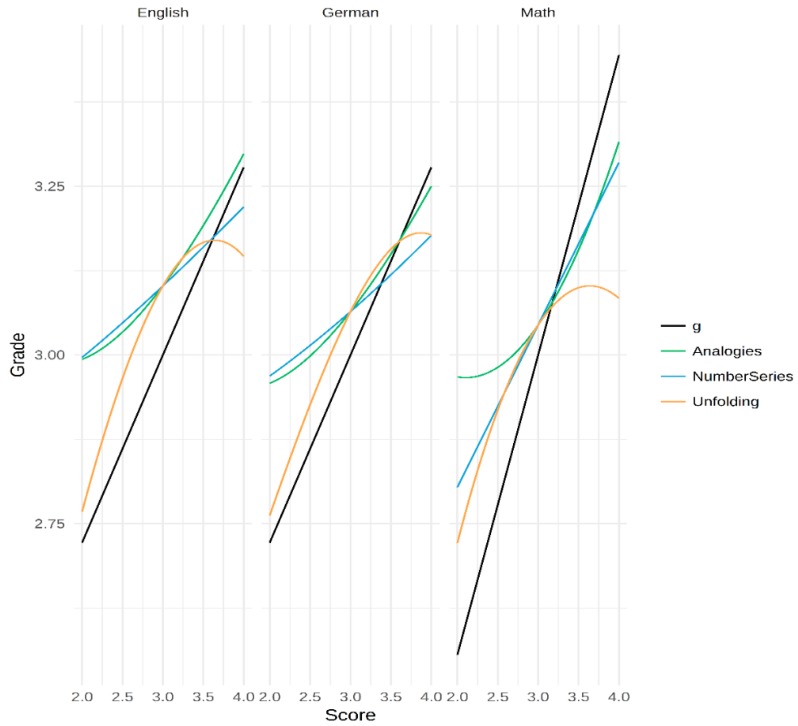
Relation between grades and ability test scores estimated from the selected models. In the case of several predictors, the levels for the respective other predictors were fixed at 3.

**Unfolding.** The Unfolding test score showed the most interesting pattern. In a linear regression, it was only significant for the prediction of math. In the polynomial regressions, this was different. The plots in Figure 4 suggest that the ability measured is related to scholastic performance mainly in the below average ability range. What this means is that the ability measured becomes less important once a threshold is met. In other words, this ability would be a requisite for passing but would not suffice to excel. Looking at the relation from the perspective of the grades, the findings could also mean that, in order to achieve truly excellent grades, other traits are of more importance. In any case, it would be beneficial to have a better understanding of the test in question. Unfolding tests as the one used here typically measure spatial and reasoning abilities. It is often assumed that such tests are good predictors of fluid intelligence (gf) because of their relatively low demand for crystalized ability (e.g., knowledge of words or numbers). Taking this into account, it could be argued that the test is a good indicator of gf. It has to be noted though that we used only one indicator of this ability. Thus, the variance due to the specific ability might be confounded with variance due to the specific test tasks. However, if we accept that this one test could be an indicator of gf, it would mean that gf is a necessary condition to master math. However, it does not suffice to truly excel, at least in terms of school grades. As noted above, other traits such as personality, motives, or interests might be more relevant. For a large sample of Swedish recruits, Lindqvist and Vestman [83] analyzed the long-term importance of ability and personality with regards to job success. They reported that cognitive ability is especially important for the initial success, while personality was important for the long-term success. It is reasonable to assume that scholastic success follows similar patterns, specifically when it comes to specific abilities or even gf. Of course, we want to emphasize that these ideas are purely speculative and should be seen as hypotheses. Further research with independent data sets is necessary to gauge the sustainability of these thoughts.

**Analogies.** Interestingly, a curvilinear relation between this ability and scholastic performance was especially pronounced in math. The analogies test used here requires a certain amount of vocabulary and reasoning. Vocabulary is a good indicator of crystallized intelligence [84]. It is reasonable to assume that the specific ability measured here helps to analyze and comprehend complex texts. In school, such texts become more and more regular even in math exams once the students enter higher grades. Thus, as argued above, it might be possible that, in order to achieve excellent grades in math, it does not suffice to understand the logic behind the analyses. It is also important to comprehend the texts within books and, maybe even more importantly, within exams [85]. This would explain why higher scores in the analogies tests are predictive of better grades.

Combining this idea with the idea of Unfolding being a specific ability test from the realm of gf tests, the current findings suggest that fluid ability might help to achieve moderate grades in math. However, in order to achieve excellent grades, above average verbal abilities might be needed. Future research could test these ideas by either analyzing or manipulating exams with regards to their reliance on text-based tasks. This finding is also in line with the mechanism derived from Spearman’s ability differentiation hypothesis. The current results show that the relation between the Analogies test scores and math performance becomes stronger in the upper regions of the ability.

On the other hand, and also with regards to Unfolding scores, the idea of grades being contaminated with the influence of other traits (e.g., interests) could be behind the observed curvilinear pattern. The results also support the notion that different specific abilities are relevant for different grade levels. It has to be noted that, given the nature of the data used, we cannot test these mechanisms directly, nor can we draw actual causal interpretations. Nevertheless, the findings are encouraging and should inspire future research to apply similar statistical approaches to longitudinal data, ideally comprising a wide range of abilities and traits. 

### 4.2. Machine Learning

In recent years, the application of machine learning algorithms has attracted more and more attention in psychology [86,87,88]. Here, we also use a machine learning approach but refer to much simpler algorithms. Still, the findings are encouraging and support the notion of furthering scientific knowledge using such complex methods. Importantly this study shows that the results and algorithms must not remain black boxes. Admittedly, applying more complex approaches like deep neural networks or support vector machines might prove even more fruitful with regards to optimizing prediction. However, in some cases, prediction alone does not suffice. Especially when it comes to predictors of scholastic performance, it seems vital to understand their relation to performance. The current analyses show that it is possible to take advantage of machine learning without giving up the possibility to derive at conclusions about possible mechanisms. Thus, we further encourage using machine learning approaches, including validation strategies, in psychological research.

### 4.3. Limitations and Outlook

The generalizability of the reported findings suffers from some limitations. Most obvious is the small sample size and the limited number of variables at hand. However, this article being part of a special issue in which everyone used the same data set outweighs this potentially damaging limitation. Due to the fact that no item level information was available, a possible alternative explanation can not be ruled out. We have emphasized above that the relation between performance and ability might not be the same across all ability levels. It is also possible that the actual ability captured within the test is not the same across score levels. This would mean that the kind of construct measured differs with differing test scores. This could also yield curvilinear relations. Measurement invariance tests would be required to test whether the same ability is captured for all score levels. Again, this is an interesting idea for future research. Another limitation comes along with the machine learning approach. While we tried to take precautions in order to avoid overfitting, no real replication was possible based on this sample. Thus, generalizing the current findings should be avoided until replications support the notion of curvilinear relations. Finally, the current paper does not really capture specific abilities. Each specific ability mentioned is only measured with one test. These tests contain variance due to *g*, the specific ability, and measurement error, but also task specific variance. Moreover, the variance accounted for by the *g*-factor often is rather substantial [36]. While this further explains the rather modest increments in *R*^2^, it also means that a strong test of the hypotheses stated above needs to operationalize each specific ability with more than one test. Related to this is a possible influence of measurement error. Typically, ability test scores are less reliable in the boundary areas. Within our analyses, this could have led to floor or ceiling effects, which in turn might also yield curvilinear relations. Especially for the Unfolding test score, this might be an alternative explanation as the form of the function does not seem to differ much across subjects. In order to rule this out, it is necessary to use tests matching the students’ abilities. Adaptive tests might be a promising approach. Despite these shortcomings, we hope that the findings inspire such replication efforts.

## 5. Conclusions

The current paper started with the idea that the world is not linear. The analyses conducted support this notion with regards to the relation between specific cognitive abilities and scholastic performance. Based on Brogden’s [25] ideas of criterion contamination and deficiency as well as Spearman’s ability differentiation hypothesis, possible mechanisms causing curvilinear relations were suggested. Using the data provided by the guest editors, we tested this idea by utilizing polynomial regressions. The findings support the idea of nonlinear relations between specific abilities and scholastic performance. Based on these results, we suggest that some cognitive abilities might simply help to achieve a moderate level of performance while others are necessary to truly excel.

## Figures and Tables

**Table 1:** Means, standard deviations, and correlations with confidence intervals.

Variable	*M*	*SD*	1	2	3	4	5	6	7	8	9	10	11
1. Age	16.03	1.49											
2. Unfolding	9.31	4.26	0.12										
			[−0.01, 0.25]										
3. Unfolding scaled	3.00	1.00	0.12	1.00 **									
			[−0.01, 0.25]	[1.00, 1.00]									
4. Analogies	8.21	3.80	0.31 **	0.33 **	0.33 **								
			[0.19, 0.43]	[0.21, 0.44]	[0.21, 0.44]								
5. Analogies scaled	3.00	1.00	0.31 **	0.33 **	0.33 **	1.00 **							
			[0.19, 0.43]	[0.21, 0.44]	[0.21, 0.44]	[1.00, 1.00]							
6. Number Series	8.26	4.01	0.21 **	0.39 **	0.39 **	0.39 **	0.39 **						
			[0.08, 0.33]	[0.27, 0.50]	[0.27, 0.50]	[0.27, 0.50]	[0.27, 0.50]						
7. Number Series scaled	3.00	1.00	0.21 **	0.39 **	0.39 **	0.39 **	0.39 **	10.00 **					
			[0.08, 0.33]	[0.27, 0.50]	[0.27, 0.50]	[0.27, 0.50]	[0.27, 0.50]	[1.00, 1.00]					
8. Factor Score (*g*)	−0.00	0.81	0.28 **	0.71 **	0.71 **	0.72 **	0.72 **	0.85 **	0.85 **				
			[0.15, 0.39]	[0.64, 0.77]	[0.64, 0.77]	[0.64, 0.77]	[0.64, 0.77]	[0.80, 0.88]	[0.80, 0.88]				
9. Factor Score (*g*) scaled	3.00	1.00	0.28 **	0.71 **	0.71 **	0.72 **	0.72 **	0.85 **	0.85 **	1.00 **			
			[0.15, 0.39]	[0.64, 0.77]	[0.64, 0.77]	[0.64, 0.77]	[0.64, 0.77]	[0.80, 0.88]	[0.80, 0.88]	[1.00, 1.00]			
10. Grade German	3.91	0.94	0.23 **	0.22 **	0.22 **	0.24 **	0.24 **	0.19 **	0.19 **	0.28 **	0.28 **		
			[0.10, 0.35]	[0.09, 0.34]	[0.09, 0.34]	[0.11, 0.36]	[0.11, 0.36]	[0.06, 0.32]	[0.06, 0.32]	[0.15, 0.40]	[0.15, 0.40]		
11. Grade English	3.74	0.94	0.19 **	0.13	0.13	0.27 **	0.27 **	0.22 **	0.22 **	0.27 **	0.27 **	0.54 **	
			[0.06, 0.32]	[−0.00, 0.26]	[−0.00, 0.26]	[0.15, 0.39]	[0.15, 0.39]	[0.09, 0.34]	[0.09, 0.34]	[0.14, 0.39]	[0.14, 0.39]	[0.44, 0.63]	
12. Grade Sports	5.05	0.72	−0.01	−0.01	−0.01	0.10	0.10	0.03	0.03	0.05	0.05	0.16 *	0.11
			[−0.15, 0.12]	[−0.14, 0.13]	[−0.14, 0.13]	[−0.04, 0.22]	[−0.04, 0.22]	[−0.11, 0.16]	[−0.11, 0.16]	[−0.09, 0.18]	[−0.09, 0.18]	[0.02, 0.28]	[−0.02, 0.24]

Note. * indicates *p* < 0.05; ** indicates *p* < 0.01. *M* and *SD* are used to represent mean and standard deviation, respectively. Values in square brackets indicate the 95% confidence interval for each correlation. The confidence interval is a plausible range of population correlations that could have caused the sample correlation [70].

**Table 2:** Regression results using the different grades as criteria and the factor score (*g*) as predictor.

Subject	Predictor	*b*	*b* 95% CI [*LL*, *UL*]	*β*	*β* 95% CI [*LL*, *UL*]	*r*	*R*^2^	*Adj. R*^2^
Math								
	Factor Score (*g*)	0.44 **	[0.32, 0.56]	0.44	[0.32, 0.56]	0.44 **		
							*R*^2^ = 0.198 **	*0.194*
							95% CI [0.11, 0.29]	
German								
	Factor Score (*g*)	0.28 **	[0.15, 0.41]	0.28	[0.15, 0.41]	0.28 **		
							*R*^2^ = 0.077 **	0.073
							95% CI [0.02, 0.15]	
English								
	Factor Score (*g*)	0.27 **	[0.14, 0.40]	0.27	[0.14, 0.40]	0.27 **		
							*R*^2^ = 0.074 **	*0.070*
							95% CI [0.02, 0.15]	
Sports								
	Factor Score (*g*)	0.05	[−0.09, 0.18]	0.05	[−0.09, 0.18]	0.05		
							*R*^2^ = 0.002	*−0.002*
							95% CI [0.00, 0.03]	

Note. * indicates *p* < 0.05; ** indicates *p* < 0.01. A significant *b*-weight indicates the *β*-weight and semi-partial correlation are also significant. *b* represents unstandardized regression weights; *β* indicates the standardized regression weights; *r* represents the zero-order correlation. *LL* and *UL* indicate the lower and upper limits of a confidence interval, respectively. *Adj. R*^2^ represents the amount of explained variance adjusted for sample size and number of predictors.

**Table 3:** Regression results using the different grades as criteria and the specific abilities as predictors.

Subject	Predictor	*b*	*b* 95% CI [*LL*, *UL*]	*β*	*β* 95% CI [*LL*, *UL*]	*sr*^2^	*sr*^2^ 95% CI [*LL*, *UL*]	*r*	*R*^2^	*Adj. R*^2^
Math										
	Unfolding	0.15 *	[0.02, 0.28]	0.15	[0.02, 0.28]	0.02	[−0.01, 0.05]	0.31 **		
	Analogies	0.22 **	[0.08, 0.35]	0.22	[0.08, 0.35]	0.04	[−0.01, 0.08]	0.35 **		
	Number Series	0.22 **	[0.08, 0.35]	0.22	[0.08, 0.35]	0.04	[−0.01, 0.08]	0.36 **		
									*R*^2^ = 0.200 **	*0.189*
									95% CI [0.11, 0.28]	
German										
	Unfolding	0.14	[−0.01, 0.28]	0.14	[−0.01, 0.28]	0.01	[−0.02, 0.05]	0.22 **		
	Analogies	0.16 *	[0.02, 0.31]	0.16	[0.02, 0.31]	0.02	[−0.02, 0.06]	0.24 **		
	Number Series	0.08	[−0.07, 0.22]	0.08	[−0.07, 0.22]	0.00	[−0.01, 0.02]	0.19 **		
									*R*^2^ = 0.083 **	*0.071*
									95% CI [0.02, 0.15]	
English										
	Unfolding	0.01	[−0.14, 0.15]	0.01	[−0.14, 0.15]	0.00	[−0.00, 0.00]	0.13		
	Analogies	0.22 **	[0.08, 0.36]	0.22	[0.08, 0.36]	0.04	[−0.01, 0.09]	0.27 **		
	Number Series	0.13	[−0.01, 0.28]	0.13	[−0.01, 0.28]	0.01	[−0.02, 0.04]	0.22 **		
									*R*^2^ = 0.089 **	*0.077*
									95% CI [0.02, 0.16]	
Sports										
	Unfolding	−0.04	[−0.19, 0.11]	−0.04	[−0.19, 0.11]	0.00	[−0.01, 0.01]	−0.01		
	Analogies	0.11	[−0.04, 0.26]	0.11	[−0.04, 0.26]	0.01	[−0.02, 0.04]	0.10		
	Number Series	−0.00	[−0.15, 0.15]	−0.00	[−0.15, 0.15]	0.00	[−0.00, 0.00]	0.03		
									*R*^2^ = 0.011	*−0.003*
									95% CI [<0.01, 0.04]	

Note. * indicates *p* < 0.05; ** indicates *p* < 0.01. A significant *b*-weight indicates the *β*-weight and semi-partial correlation are also significant. *b* represents unstandardized regression weights; *β* indicates the standardized regression weights; *sr*^2^ represents the semi-partial correlation squared; *r* represents the zero-order correlation. *LL* and *UL* indicate the lower and upper limits of a confidence interval, respectively. *Adj. R*^2^ represents the amount of explained variance adjusted for sample size and number of predictors.

**Table 4:** Summaries of the best fitting linear and polynomial equations.

**Predictor**	**Criterion**	**Degree**			**Adjusted *R*^2^**	**RMSE**
***g*-factor**						
	Math	1			0.194	0.644
	German	1			0.073	0.687
	English	1			0.070	0.648
**Specific Ability Test Scores**		**Unfolding**	**Analogies**	**Number Series**		
	Math	2	2	1	0.220	0.611
	German	3	0.5	0.5	0.083	0.637
	English	5	0.5	1.5	0.091	0.607

## Footnotes

1 The OSF link to this paper is: https://osf.io/g69ke/?view_only=9e35c20578904c37a418a7d03218dbff. Here, you can find the R code for these analyses, the data set, as well as further analyses mentioned.

2 The OSF link to this paper is: https://osf.io/g69ke/?view_only=9e35c20578904c37a418a7d03218dbff. Here, you can find the R code for these analyses, the data set, as well as further analyses mentioned.
